# 

**Published:** 1889-05

**Authors:** 


### CONTENTS.

***MISCELLANEOUS.*** PAGE

Questions asked by Students during a Course of Lectures on *The Organon.* Prof. Gee, . . . 169
Repetition of The Remedy. Translation by Dr. A. McNeil, . . . 171
Iris Versicolor. Dr. A. McNeil, . . . 173
Constipation of Berberis Vulgaris. Dr. John L. Ferson, . . . 174
Proceedings of The Lippe Society. Dr. Geo. H. Clark, Sec., . . . 177
Diphtheria. Dr. Horace Still, . . . 182
Organon Society of Boston. Dr. S. A. Kimball, Sec., . . . 187
Verifications. Dr. A. H. Birdsall, . . . 189
Clinical Cases. Dr. E. W. Berridge, . . . 192
Fracture of a Rib with Aggravation and Relief from Hypericum. Dr. B. L. B. Baylies, . . . 198
The April Number, . . . 198
Clinical Cases. Dr. C. N. Payne, . . . 199
Polypus of Rectum. Mr. Alfred Heath, F. L. S., . . . 200
Croup. Dr. L. P. Foster, . . . 201
Prof. Morgan's Lectures, . . . 202
Diarrhœa of Consumptives. Dr. F. L. Griffith, . . . 202
Intercurrent Remedies for Chronic Diseases. Translation by Dr. F. H. Lutze, . . . 203
Case for Counsel. Dr. George H. Clark, . . . 205
Verifications. Dr. Oran W. Smith, . . . 207
Nux Vomica. Dr. F. L. Griffith, . . . 210
An Unnoticed Symptom of Ipecacuanha, clinically verified by Dr. Mossa, . . . 211
Allopathic Ignorance and Arrogance. Dr. B. Fincke, . . . 212
Croup, Bœnninghausen and McNeil. Dr. P. P. Wells, . . . 213

***BOOK NOTICES AND REVIEWS.***

Alcoholisme et Criminalité. Par Dr. Gallavardin, . . . 215

International Medical Annual, . . . 215

***NOTES AND NOTICES.***

Died—Removals—Partnership—Married—Southern Journal of Homœopathy—A Good Opening—The Medical Annual—The International Hahnemannian Association—The Journal of Homœopathics, . . . 215

